# MRI textural plasticity in limbic gray matter associated with clinical response to electroconvulsive therapy for psychosis

**DOI:** 10.1038/s41380-024-02755-7

**Published:** 2024-09-26

**Authors:** Eugenie Choe, Minah Kim, Sunah Choi, Harin Oh, Moonyoung Jang, Sunghyun Park, Jun Soo Kwon

**Affiliations:** 1 Department of Neuropsychiatry, Seoul National University Hospital, Seoul, Republic of Korea; 2 Department of Psychiatry, Seoul National University College of Medicine, Seoul, Republic of Korea; 3 Department of Clinical Pharmacology and Therapeutics, Seoul National University College of Medicine, Seoul, Republic of Korea; 4 Department of Brain and Cognitive Sciences, Seoul National University College of Natural Sciences, Seoul, Republic of Korea; 5 Department of Psychiatry, Hanyang University Hospital, Seoul, South Korea; 6 Department of Psychiatry, Hanyang University College of Medicine, Seoul, South Korea; 7 Institute of Human Behavioral Medicine, SNU-MRC, Seoul, Republic of Korea

**Keywords:** Schizophrenia, Neuroscience

## Abstract

Electroconvulsive therapy (ECT) is effective against treatment-resistant psychosis, but its mechanisms remain unclear. Conventional volumetry studies have revealed plasticity in limbic structures following ECT but with inconsistent clinical relevance, as they potentially overlook subtle histological alterations. Our study analyzed microstructural changes in limbic structures after ECT using MRI texture analysis and demonstrated a correlation with clinical response. 36 schizophrenia or schizoaffective patients treated with ECT and medication, 27 patients treated with medication only, and 70 healthy controls (HCs) were included in this study. Structural MRI data were acquired before and after ECT for the ECT group and at equivalent intervals for the medication-only group. The gray matter volume and MRI texture, calculated from the gray level size zone matrix (GLSZM), were extracted from limbic structures. After normalizing texture features to HC data, group-time interactions were estimated with repeated-measures mixed models. Repeated-measures correlations between clinical variables and texture were analyzed. Volumetric group-time interactions were observed in seven of fourteen limbic structures. Group-time interactions of the normalized GLSZM large area emphasis of the left hippocampus and the right amygdala reached statistical significance. Changes in these texture features were correlated with changes in psychotic symptoms in the ECT group but not in the medication-only group. These findings provide in vivo evidence that microstructural changes in key limbic structures, hypothetically reflected by MRI texture, are associated with clinical response to ECT for psychosis. These findings support the neuroplasticity hypothesis of ECT and highlight the hippocampus and amygdala as potential targets for neuromodulation in psychosis.

## Introduction

Although electroconvulsive therapy (ECT) is an important treatment modality for treatment-resistant psychotic disorders, its therapeutic mechanism remains elusive. The putative biological mechanisms of ECT include structural neuroplasticity and neuroinflammation [1]. These hypotheses are supported by animal studies demonstrating significant histopathological changes after electroconvulsive stimulation, including neurogenesis [2], synaptogenesis [3], angiogenesis [4], gliogenesis [5], and axonal modifications [6], particularly in medial temporal regions such as the dentate gyrus [7–9].

In human studies, structural MRI has been utilized to explore ECT-induced brain alterations [10–13]. These studies revealed significant structural changes within limbic regions, such as the hippocampus, amygdala, and parahippocampal gyrus [10, 11, 14, 15]. These regions play crucial roles in the limbic system and are associated with the pathophysiology of positive [16, 17] and negative [16, 18, 19] psychotic symptoms. Although such studies suggest a potential link between ECT-induced structural changes in the limbic regions and improvements in psychotic symptoms, the evidence is not yet conclusive, with varying results across studies. While some studies have identified correlations between structural changes and symptom modifications after ECT [11, 12, 20, 21], others have not found a significant association [10, 11, 14].

Previous research on structural alterations following ECT predominantly focused on macrostructural alterations, primarily volumetric changes. However, changes in gray matter volume measured by MRI volumetry may reflect a variety of underlying neurological phenomena. For example, an increase in water content or an increase in arterial blood flow within the gray matter has been proposed as a possible factor that, in addition to histopathological changes, may have an impact on gray matter volume. Although MRI modalities other than conventional T1-weighted MR images, such as T2-weighted MR images [22] or arterial spin labeling MR images [23, 24], may complement these methods to provide more insight into the mechanism of volumetric alterations related to ECT, conventional MR volumetry is still limited with regard to fully discriminating the subtle neurological impacts of ECT.

To elucidate whether the histopathological plasticity observed in animal studies is a central therapeutic mechanism of ECT in the human brain, it is crucial to refine structural measures to be more reflective of microstructural alterations. T1 relaxation on MRI has shown potential sensitivity in the identification of histological tissue characteristics [25–28]. For example, a study on histological correlates of MRI parameters in temporal lobe epilepsy revealed that, among quantitative relaxometry and diffusion parameters, T1 may be a predictor of neuron density for both large and small neurons [25]. Accordingly, measures that represent more detailed T1 signal information per voxel and thereby more sensitively reflect histopathological traits are needed, since such information is largely lost in macrostructural measures. Texture analysis, a mathematical method for quantifying the internal structure of voxels [29], has been proposed for the extraction of radiomic features that more accurately depict histopathological traits than macrostructural measures across various neurological conditions, including brain tumors [30], Alzheimer’s disease [31], and psychosis spectrum disorders [32]. In terms of ECT in psychotic disorders, one study demonstrated the implication of radiomic features beyond volume by reporting that MRI radiomic features at baseline may predict response to ECT [33]. Nevertheless, as the study used an agnostic machine learning approach without an a priori hypothesis and primarily focused on prediction from baseline data, it has limitations in explicitly explaining which radiomic feature brain regions exhibit alterations following ECT.

In this study, we aimed to leverage texture analysis techniques to characterize microstructural plasticity in the limbic system induced by ECT in patients with psychotic disorders and to determine whether this phenomenon is related to clinical response. We hypothesized that ECT induces microstructural plasticity measurable by MRI texture in the limbic structures of psychosis patients and that these changes correlate with improvements in psychotic symptoms.

## Materials and methods

### Study participants and clinical assessment

The study sample consisted of three groups: patients with schizophrenia or schizoaffective disorder who were treated with both ECT and medication, patients with schizophrenia or schizoaffective disorder treated with medication only, and healthy controls (HCs). Patients in the ECT group were recruited from inpatients admitted to Seoul National University Hospital for ECT. Patients in the medication-only group were enrolled from Seoul National University Hospital and were recruited to have comparable age, sex, and duration of illness with patients in the ECT group. The psychotic symptom severity of the participants in each patient group was assessed using Positive and Negative Symptom Scale (PANSS) and Clinical Global Impression-Severity Scale (CGI-S). HCs were recruited via Internet advertisements and screened for Axis I disorders using SCID-I Non-Patient Edition (SCID-NP). Individuals with any current or past psychiatric illness or any family history of psychiatric disorders were excluded from the HC group. Common exclusion criteria for all groups included (1) past history of ECT, (2) significant personality disorder, (3) substance use disorder other than nicotine, and (4) presence or history of significant medical/surgical illness.

All participants received thorough explanations of the study and provided written informed consent (IRB no. H-1706-111-860 and H-1110-009-380). This study was conducted in accordance with the Declaration of Helsinki and was approved by the Institutional Review Board of Seoul National University Hospital (IRB no. H-2402-088-1511).

### ECT procedure

For patients in the ECT group, brief-pulse bitemporal stimuli were administered 2 to 3 times a week using an integrated SPECTRUM 5000Q instrument (MECTA Corporation, Portland, OR, USA). The number of ECT sessions was decided according to each patient’s symptomatic improvement (mean number of ECT sessions, 13.31 ± 3.05).

### MRI acquisition, preprocessing, and selection of regions of interest

T1-weighted MR data were acquired using a Siemens 3T Magnetom Trio Tim syngo MR scanner and a 3D MPRAGE sequence, with a repetition time of 1670 ms, an echo time of 1.89 ms, and a voxel size of 1 × 0.98 × 0.98 mm^3^. After visual inspection of the acquired images, FreeSurfer (version 6.0) was used for further processing. Motion correction, nonbrain tissue removal, and Talairach transformation were followed by cortical parcellation and subcortical segmentation. Among cortical ROIs from the Desikan-Killiany atlas and subcortical ROIs from the atlas published in Fischl et al. [34], those constituting the limbic structure were subjected to further analyses. The corresponding brain regions were the hippocampus, amygdala, accumbens area, ventral diencephalon, anterior cingulate gyri, and parahippocampal gyri. The gray matter volume of each ROI was extracted from the FreeSurfer output.

### Texture feature calculation and selection

The texture features of each ROI were calculated using the gray level size zone matrix (GLSZM) [35], which quantifies the number of connected voxels that share the same gray-level intensity. Unlike other second-order texture matrices, GLSZMs are rotation independent, making them suitable for processing neuroimaging data, which may involve image rotations. Features derived from GLSZM were calculated using Python 3 and the Python package PyRadiomics 3.1.0 [36]. A total of sixteen GLSZM features were identified (Supplementary Table 1).

To address the high-dimensionality problem, representative features were selected from the sixteen features. Before feature selection, the values of each GLSZM feature in the HC group were normalized through z-transformation, and principal component analysis (PCA) was performed on the normalized feature values. As the PCA results revealed that five principal components explained more than 90% of the variance, the top contributing features of each of the five components were selected and further analyzed. The five selected components were high gray level zone emphasis, large area emphasis, small area emphasis, gray level variance, and large area low gray level emphasis (Table 1). In the patient groups, the values of each selected GLSZM feature were normalized based on the data from the HC group.

**Table 1:** List of selected gray-level size zone matrix (GLSZM) texture features.

Feature name	Formula [35, 36]	Description [35, 36]
High gray level zone emphasis	${HGLZE}=\,\frac{{\sum }_{i=1}^{{N}_{g}}{\sum }_{j=1}^{{N}_{s}}{{\bf{P}}}(i,j){i}^{2}}{{N}_{z}}$	Distribution of the higher gray-level values
Large area emphasis	${LAE}=\,\frac{{\sum }_{i=1}^{{N}_{g}}{\sum }_{j=1}^{{N}_{s}}{{\bf{P}}}(i,j){j}^{2}}{{N}_{z}}$	Distribution of large area size zones
Small area emphasis	${SAE}=\,\frac{{\sum }_{i=1}^{{N}_{g}}{\sum }_{j=1}^{{N}_{s}}\frac{{{\bf{P}}}(i,j)}{{j}^{2}}}{{N}_{z}}$	Distribution of small size zones
Gray level variance	${GLV}=\,\mathop{\sum}\limits _{i=1}^{{N}_{g}}\mathop{\sum}\limits _{j=1}^{{N}_{s}}p(i,j){(i-\mu )}^{2}$	Variance in gray level intensities for the zones
Large area low gray level emphasis	${LALGLE}=\,\frac{{\sum }_{i=1}^{{N}_{g}}{\sum }_{j=1}^{{N}_{s}}\frac{{{\bf{P}}}(i,j){j}^{2}}{{i}^{2}}}{{N}_{z}}$	Proportion in the image of the joint distribution of larger size zones with lower gray-level values

${N}_{g}$ Number of discrete intensity values in the image.

${N}_{s}$ Number of discrete zone sizes in the image.

${N}_{z}$ Number of zones in the ROI.

${{\bf{P}}}(i,j)$ Size zone matrix.

$p(i,j)$ Normalized size zone matrix.

### Statistical analyses

Statistical analyses were performed using R version 4.3.2. Statistical significance was set as *p* < 0.05, and false discovery rate (FDR) correction was implemented for multiple comparisons correction.

Group comparisons of demographic and clinical variables at baseline were conducted using analysis of variance (ANOVA), independent *t* tests, or chi-square tests, as appropriate. Tukey tests were performed as *post hoc* tests for ANOVA.

To evaluate whether the ECT group showed significant differences in the volume or texture features of each limbic ROI after ECT compared to the medication-only group, group-time interactions of volume or texture features of each ROI were estimated using mixed models for repeated measures. Age, sex, and olanzapine-equivalent dose of antipsychotics [37] were included as covariates in each model.

Repeated-measures correlation analyses were performed on the texture feature–ROI pairs that showed significant group–time interactions to assess correlations with clinical response. The clinical variables of interest were the PANSS total score, PANSS subscale score (positive, negative, and general), and CGI-S score.

## Results

### Clinical and demographic characteristics of the study participants

A total of 36 patients who were treated with both ECT and medication, 27 patients who received medication alone, and 70 HCs were included in the study. Data from the HCs were used as a reference.

Table 2 summarizes the demographic and clinical characteristics of each group. The HCs were significantly younger than the subjects in the two patient groups, with no significant differences in age between ECT and medication-only groups (*F* = 16.17, *p* < 0.001). There were also no significant differences in the duration of illness between the two patient groups. The ECT group had higher doses of antipsychotics (*t* = 5.45, *p* < 0.001) and, at baseline, higher PANSS total (*t* = 3.59, *p* = 0.001), PANSS positive subscale (*t* = 2.50, *p* = 0.015), PANSS general subscale (*t* = 3.96, *p* < 0.001), and CGI-S (*t* = 3.65, *p* = 0.001) scores. At follow-up, no significant difference in symptom severity was observed.

**Table 2:** Demographic and clinical characteristics of the participants.

		Group			Statistical analysis^a^		*Post hoc* statistical analysis^b^		
		ECT (*n* = 36)	MED (*n* = 27)	HC (*n* = 70)	F or T or χ2	*p*	ECT - MED	ECT - HC	MED - HC
Age		32.94 (6.89)	34.00 (9.20)	25.49 (8.12)	16.17	<0.001**	0.864	<0.001**	<0.001**
Sex (male/female)		19/17	17/10	39/31	0.68	0.712	–	–	–
Duration of illness		158.31 (80.04)	149.41 (74.04)	–	0.45	0.654	–	–	–
Mean dose of antipsychotic exposure^c^		44.27 (21.91)	19.94 (8.59)	–	5.45	<0.001**	–	–	–
Number of ECT sessions		13.31 (3.05)	–	–	–				
Duration of follow-up (days)		50.11 (42.15)	46.67 (38.26)	–	2.00	0.740	–	–	–
Symptom severity at baseline									
PANSS total		77.25 (20.56)	61.33 (12.00)	–	3.59	0.001**	–	–	–
PANSS positive subscale		20.56 (6.45)	16.93 (4.51)	–	2.5	0.015*	–	–	–
PANSS negative subscale		19.25 (6.48)	16.00 (6.46)	–	1.97	0.053	–	–	–
PANSS general subscale		37.61 (10.69)	28.41 (6.46)	–	3.96	<0.001**	–	–	–
CGI-severity		5.22 (0.72)	4.52 (0.80)	–	3.65	0.001**	–	–	–
Symptom severity at follow-up									
PANSS total		60.31 (18.76)	58.11 (15.65)	–	0.49	0.624	–	–	–
PANSS positive subscale		14.81 (5.83)	19.07 (10.99)	–	−2.0	0.051	–	–	–
PANSS negative subscale		16.36 (5.50)	16.07 (6.71)	–	0.19	0.853	–	–	–
PANSS general subscale		29.67 (8.91)	27.19 (7.82)	–	1.20	0.254	–	–	–
CGI-severity		4.22 (0.96)	4.37 (0.74)	–	−0.67	0.508	–	–	–

*ECT* patients treated with both electroconvulsive therapy and medication, *MED* patients treated with medication only, *HCs* healthy controls, *PANSS* Positive and Negative Syndrome Scale, *CGI-S* Clinical Global Impression-Severity.

^a^Analysis of variance or independent t test; χ2 analysis for categorical data.

^b^*Post hoc* Tukey honestly significant difference (HSD) adjusted *p* value for variables that showed significant between-group differences in analysis of variance.

^c^Given in olanzapine-equivalent dose [37] Data are given as means (standard deviations).

*The statistical significance is *p* < 0.05; **the statistical significance is *p* < 0.005.

### Group differences in volumetric changes for each limbic ROI

At baseline, no significant group differences in the volume of any of the limbic structures were detected. However, mixed models for repeated measures, with age, sex, olanzapine-equivalent dose of antipsychotics and estimated intracranial volume included as covariates, revealed significant group–time interactions in volumes of the bilateral hippocampi, bilateral amygdala, bilateral caudal anterior cingulate gyri, and the left rostral anterior cingulate gyrus (Table 3).

**Table 3:** Limbic regions of interest (ROIs) that showed significant group–time interactions in texture and volume.

	Normalized GLSZM large area emphasis at baseline		Change in normalized GLSZM large area emphasis at baseline		Group-time interaction
	ECT	MED	ECT	MED	*p*-FDR
Left hippocampus	−1.27 (1.16)	−0.82 (1.00)	0.58 (0.82)	−0.22 (0.71)	0.008
Right amygdala	−0.70 (0.89)	−0.43 (1.21)	0.55 (0.94)	−0.40 (1.13)	0.016

	Volume at baseline (mm^3^)		Change in volume (mm^3^)		Group-time interaction
	ECT	MED	ECT	MED	*p*-FDR
Left hippocampus	3765.6 (402.6)	3860.0 (367.8)	198.5 (142.9)	11.51 (114.8)	<0.001
Right hippocampus	4029.8 (485.9)	4114.7 (366.0)	152.7 (179.7)	−0.2 (135.2)	0.003
Left amygdala	1522.8 (196.1)	1557.6 (187.6)	108.5 (121.3)	−16.1 (107.7)	<0.001
Right amygdala	1606.2 (202.7)	1663.9 (232.1)	135.5 (125.8)	−13.8 (105.7)	<0.001
Left caudal anterior cingulate gyrus	1585.2 (410.7)	1671.3 (413.8)	59.2 (78.7)	−5.7 (120.2)	0.039
Right caudal anterior cingulate gyrus	1841.3 (432.8)	1936.7 (498.2)	60.3 (73.8)	−8.0 (77.9)	0.004
Left rostral anterior cingulate gyrus	2404.1 (509.0)	2544.4 (435.7)	79.1 (128.6)	−32.2 (110.1)	0.006

Data are presented as means (standard deviations).

*ECT* patients treated with both electroconvulsive therapy and medication, *MED* patients treated with medication only, *FDR* false discovery rate.

### Group differences in texture changes for each limbic ROI

Mixed models for repeated measures, with age, sex, and olanzapine-equivalent dose of antipsychotics included as covariates, showed significant group–time interactions in the normalized GLSZM large area emphasis of the left hippocampus and the right amygdala.

**Fig. 1:**
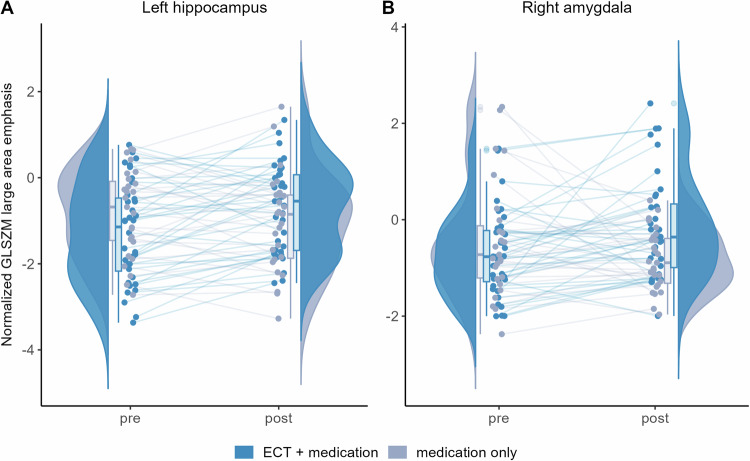
Significant group-time interactions in the normalized gray-level size zone matrix (GLSZM) large area emphasis of the left hippocampus and the right amygdala. Group-time interactions in the normalized gray-level size zone matrix (GLSZM) large area emphasis in (**A**) the left hippocampus and (**B**) the right amygdala in patients treated with both electroconvulsive therapy (ECT) and medication (blue) and patients treated with medication only (gray) are shown. The rainclouds represent the distribution of the normalized GLSZM large area emphasis in (**A**) the left hippocampus and (**B**) the right amygdala at each time point in the ECT group (blue) and the medication-only group (gray), respectively. Lines in lighter shades indicate data from individual participants in the ECT group (blue) and the medication-only group (gray). ECT electroconvulsive therapy, GLSZM gray level size zone matrix.

At baseline, there were no statistically significant group differences in the normalized GLSZM large area emphasis between the ECT and medication-only groups. At follow-up, the feature values of both the left hippocampus and the right amygdala tended to approach zero after ECT, indicating values closer to those of HCs (Fig. 1). Figure 1 also suggests that patients with greater GLSZM large area emphasis in the left hippocampus than HCs at baseline tended to have decreased GLSZM large area emphasis after ECT that approached the reference value.

### Repeated-measures correlation between texture and clinical variables

**Fig. 2:**
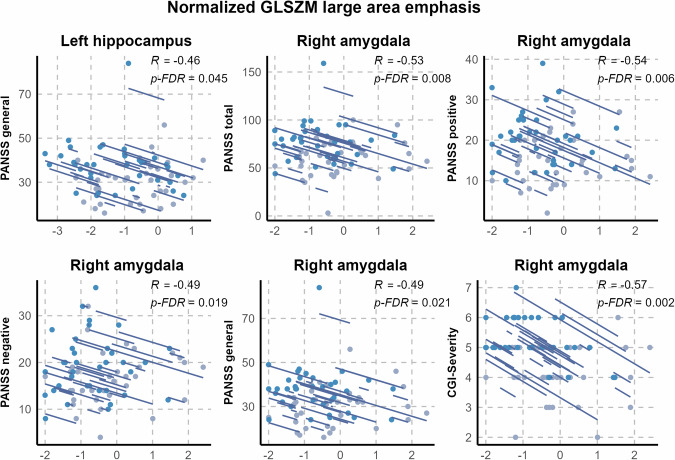
Statistically significant repeated-measures correlations between clinical variables and normalized gray-level size zone matrix (GLSZM) large area emphasis of the left hippocampus and the right amygdala in patients treated with both electroconvulsive therapy and medication. Repeated-measures correlation coefficients and false discovery rate (FDR)-adjusted *p* values are shown. The blue dots represent data before ECT; the gray dots represent data after ECT. PANSS Positive and Negative Syndrome Scale, CGI-S Clinical Global Impression Severity, FDR False Discovery Rate, GLSZM gray level size zone matrix.

Overall, normalized GLSZM large area emphasis and clinical variables correlated negatively in the ECT group. Regarding the left hippocampus, only the PANSS general subscale remained a statistically significant correlate of the texture feature after multiple comparison correction. For the right amygdala, the texture feature correlated with all clinical variables of interest (PANSS total score, PANSS subscales, and CGI severity) (Fig. 2 and Supplementary Fig. 1).

In the medication-only group, no significant correlation was observed between texture features and clinical variables.

## Discussion

In this study, we aimed to investigate MRI textural plasticity in the limbic system induced by ECT in patients with psychotic disorders and its relevance to clinical response in psychosis patients. The microstructure of limbic structures, which is hypothetically reflected by texture features in in vivo MRI, was quantified using features from GLSZM. Significant group-time interactions between two patient groups, i.e., patients treated with both ECT and medication and patients treated with medication only, were observed in the large area emphasis feature of the left hippocampus and the right amygdala. Repeated measure correlation analyses revealed that for patients treated with both ECT and medication, a change in large area emphasis of the left hippocampus correlated with a change in the PANSS general subscale score and that a change in the right amygdala correlated with changes in all PANSS subscales and CGI scores. In comparison to a previous agnostic radiomic approach that sought to predict ECT response in psychotic disorders [33], this study provides more insight into the specific textural alterations that occur in certain brain regions after ECT, as well as evidence from an in vivo human study that neuroplasticity in key limbic structures is associated with therapeutic effects of ECT for psychotic disorders.

Although not all limbic regions that exhibited volumetric changes after ECT showed statistically significant alterations in texture, the regions that did show significant changes in texture features were the core brain regions that have been posited to be the most affected by ECT. Among the five selected GLSZM features, large area emphasis was shown to have significant group–time interactions in both the right amygdala and the left hippocampus. This finding suggests that large area emphasis might be a texture feature that is sensitive to ECT-induced microstructural changes in psychosis patients. Mathematically, large area emphasis quantifies the distribution of large area size zones in a GLSZM; therefore, a larger large area emphasis indicates a coarser structure. Although this finding should be interpreted with caution because its direct correlation with histological properties has yet to be established, one putative explanation is that changes in the coarseness of the gray matter may reflect reorganization of the neuroarchitecture. Evidence from postmortem studies suggests that schizophrenia patients have altered hippocampal and amygdalar histology, such as a decreased total number of neurons [38] or extracellular matrix-glial abnormalities [39]. Perturbation in chronically altered histological structures induced by ECT may increase the coarseness of gray matter [32]. Overall, these results support the neuroplasticity hypothesis that ECT may induce microstructural histological changes in the human brain.

The hippocampus is the most consistently mentioned limbic structure that shows increased volume after ECT [14, 15, 40, 41]. The results of the current study are in line with the literature, as significant group-time interactions in hippocampal volume were detected. The current study also showed that in the left hippocampus, alterations in the normalized GLSZM large area emphasis were correlated with improvements in the PANSS general subscale score, while no significant correlation was observed with the positive or negative psychotic symptom subscales. This result may indicate that plasticity in the hippocampus is more closely related to improvement in general psychopathology rather than psychotic symptoms per se. Nevertheless, numerous meta-analyses, albeit specifically investigating depression, have concluded that volume changes in the hippocampus may not be associated with clinical response [40, 41]. One possible explanation for this discrepancy is that volume changes and texture changes differ in the breadth of the neurological phenomena that they reflect. This explanation is in part supported by the finding that some studies have reported a weak, negative correlation between an increase in hippocampal volume and improvement in depressive symptoms, which is counterintuitive [40]. Since gray matter volume on MRI can be affected by diverse neurological changes, the additive effect of such alterations might have led to inconsistent results; texture, which is regarded to be more reflective of the gray matter microstructure, would have shown a more robust effect.

In the right amygdala, the normalized GLSZM large area emphasis correlated significantly with all clinical variables of interest (PANSS total, PANSS subscales, and CGI-S). Along with the hippocampus, the amygdala has frequently been highlighted as a brain region that is modulated by ECT [42–44]. For example, a study that included both schizophrenia and major depression patients reported that the amygdala displayed transdiagnostic volumetric changes after ECT [10]. The amygdala, a pivotal structure in the mesocorticolimbic dopaminergic system, plays a crucial role in emotional and cognitive functions, affecting how people perceive, process and remember emotional and sensory information. It is also gaining attention in the context of reinforcement learning, as it is suggested to process input stimuli alongside top-down predictions and convey prediction error signals to higher cortical levels [45]. Deficiency in this process is regarded as integral to the prediction-error hypothesis of schizophrenia, whereby aberrations in processing prediction errors are believed to contribute to psychotic symptoms [46]. As support for the importance of the amygdala in the pathophysiology of schizophrenia under this hypothesis, previous functional MRI studies that sought to map impaired prediction error processing in schizophrenia patients reported inclusion of amygdalar structure in associated clusters. Gradin et al. [47] found that the amygdala-hippocampal complex was one of the regions that showed reduced prediction error signals and disrupted encoding of expected reward values and that the degree of signal alterations correlated with the severity of psychotic symptoms. A recent meta-analysis also revealed that schizophrenia patients exhibit reduced activity in the amygdala and other mesolimbic regions when processing prediction errors [48]. Taken together, our findings that structural changes in the amygdala correlate with clinical response to ECT provide additional evidence that the amygdala might be a key structure in both the therapeutic mechanism of ECT and the pathophysiology of psychotic symptoms. Thus, the role of the amygdala in psychotic disorders and ECT warrants further investigation.

This study has several limitations. First, as the participants in the patient groups were not randomly allocated, there was a significant difference between the two patient groups in antipsychotic drug dosage and symptom severity at baseline. This is primarily due to the observational nature of this study, as patients with more severe symptoms are more likely to be candidates for ECT. Nevertheless, we attempted to minimize the difference in symptom severity by enrolling patients with relatively severe symptoms in the medication-only group, resulting in a mean total PANSS score of 61.33 (Table 2). Additionally, the mean olanzapine-equivalent dose of antipsychotics was added as a covariate in the mixed models. Second, our texture analyses focused on GLSZM, yet there are many other possible radiomic features. This approach was taken with an a priori assumption that the GLSZM would be the most suitable second-order radiomic feature in structural neuroimaging studies because it is independent of rotation [35], which may occur during image processing, such as registration. It was also expected to enhance the interpretability of the results compared to those of an agnostic radiomic study in which numerous texture features were input into a machine learning model without prior hypotheses. Nevertheless, our approach may be limited in terms of its generalizability. Third, more evidence on the direct relationship between MRI texture and actual histological characteristics should be obtained for a more precise interpretation of our results.

This study investigated ECT-related textural changes in limbic structures and their clinical correlations in psychotic disorders. In accordance with the literature in which the hippocampus and the amygdala have repeatedly been suggested to be key regions of macrostructural alterations associated with ECT, our results suggest that changes in the texture of these regions correlate with clinical response. By providing evidence from an in vivo human study that ECT-related microstructural alterations have clinical relevance in psychosis patients, these findings support the neuroplasticity hypothesis of the therapeutic mechanism of ECT in psychosis. Specifically, our results underscore the importance of the hippocampus and amygdala in the therapeutic effects of ECT and, in part, suggest that these regions may be pivotal for development of targeted neuromodulation techniques that mimic the effects of ECT.

## Supplementary information

Supplementary Figure and Table

## Data Availability

Data is available upon request. Correspondence and requests for materials should be addressed to Minah Kim.
