# Supplementary material for: MRI textural plasticity in limbic gray matter associated with clinical response to electroconvulsive therapy for psychosis
Source: Mol Psychiatry. 2024 Sep 26;30(4):1453–60. doi: 10.1038/s41380-024-02755-7 (PMC11919751; doi:10.1038/s41380-024-02755-7)
Supplement: Supplementary file 1 — Supplementary Figure and Table [file 41380_2024_2755_MOESM1_ESM.docx]

**Supplementary figures**


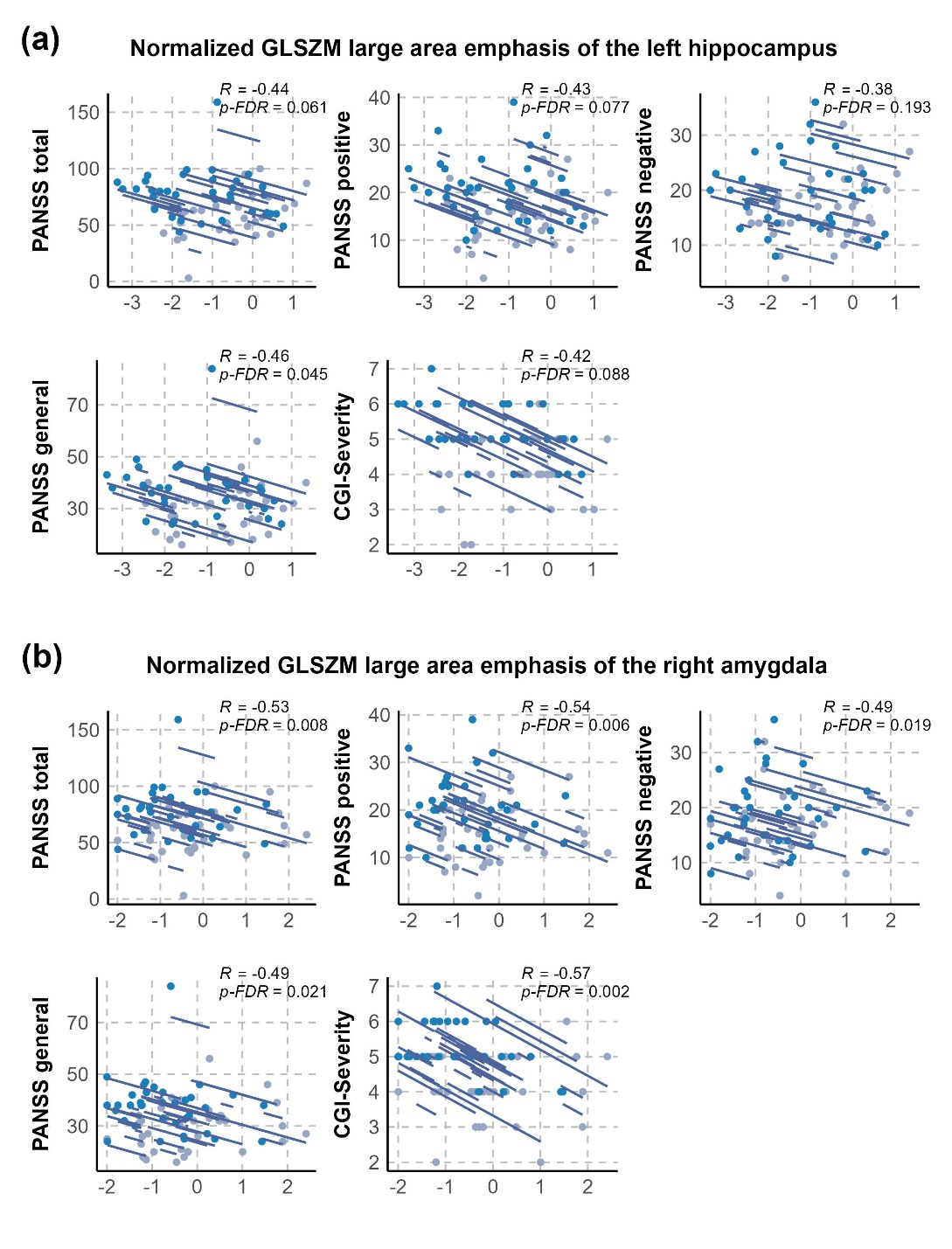


**Supplementary Figure 1.** Repeated-measures correlations between all clinical variables of interest and the normalized GLSZM large area emphasis of (a) the left hippocampus and (b) the right amygdala in patients treated with both electroconvulsive therapy and medication. Repeated-measures correlation coefficients and FDR-adjusted *p* values are shown. The blue dots represent data before ECT; the gray dots represent data after ECT.

Abbreviations: PANSS, Positive and Negative Syndrome Scale; CGI-S, Clinical Global Impression Severity; FDR, false discovery rate; GLSZM, gray level size zone matrix

**Supplementary Table 1.** List of gray level size zone matrix (GLSZM) texture features.

| Feature name | Formula [1,2] | Description [1, 2] |
| --- | --- | --- |
| Small area emphasis | $SAE= \frac{\sum_{i=1}^{N_{g}} \sum_{j=1}^{N_{s}} \frac{\mathbf{P}(i,j)}{j^{2}}}{N_{z}}$ | Distribution of large area size zones |
| Large area emphasis | $LAE= \frac{\sum_{i=1}^{N_{g}} \sum_{j=1}^{N_{s}} \mathbf{P}(i,j)j^{2}}{N_{z}}$ | Distribution of large area size zones |
| Gray level non-uniformity | $GLN= \frac{\sum_{i=1}^{N_{g}} {(\sum_{j=1}^{N_{s}} \mathbf{P}\left( i,j \right))}^{2}}{N_{z}}$ | Variability of gray-level intensity values |
| Gray level non-uniformity normalized | $GLNN= \frac{\sum_{i=1}^{N_{g}} {(\sum_{j=1}^{N_{s}} \mathbf{P}\left( i,j \right))}^{2}}{{N_{z}}^{2}}$ | Variability of gray-level intensity values |
| Size-zone non-uniformity | $SZN= \frac{\sum_{j=1}^{N_{s}} {(\sum_{i=1}^{N_{g}} \mathbf{P}\left( i,j \right))}^{2}}{N_{z}}$ | Variability of size zone volumes |
| Size-zone non-uniformity normalized | $SZNN= \frac{\sum_{j=1}^{N_{s}} {(\sum_{i=1}^{N_{g}} \mathbf{P}\left( i,j \right))}^{2}}{{N_{z}}^{2}}$ | Variability of size zone volumes |
| Zone percentage | $ZP= \frac{N_{z}}{N_{p}}$ | Ratio of number of zones and number of voxels |
| Gray level variance | $GLV= \sum_{i=1}^{N_{g}} \sum_{j=1}^{N_{s}} p(i,j){(i-\mu)}^{2}$ | Variance in gray level intensities for the zones |
| Zone variance | $ZV= \sum_{i=1}^{N_{g}} \sum_{j=1}^{N_{s}} p(i,j){(j-\mu)}^{2}$ | Variance in zone size volumes for the zones |
| Zone entropy | $ZE= -\sum_{i=1}^{N_{g}} \sum_{j=1}^{N_{s}} p(i,j)\log_{2} (p\left( i,j \right)+ \epsilon)$ | Uncertainty/randomness in the distribution of zone sizes and gray levels |
| Low gray level zone emphasis | $LGLZE= \frac{\sum_{i=1}^{N_{g}} \sum_{j=1}^{N_{s}} \frac{\mathbf{P}(i,j)}{i^{2}}}{N_{z}}$ | Distribution of lower gray-level size zones |
| High gray level zone emphasis | $HGLZE= \frac{\sum_{i=1}^{N_{g}} \sum_{j=1}^{N_{s}} \mathbf{P}(i,j)i^{2}}{N_{z}}$ | Distribution of higher gray-level values |
| Small area low gray level emphasis | $SALGLE= \frac{\sum_{i=1}^{N_{g}} \sum_{j=1}^{N_{s}} \frac{\mathbf{P}(i,j)}{i^{2}j^{2}}}{N_{z}}$ | Proportion of the joint distribution of smaller size zones with lower gray-level values |
| Small area high gray level emphasis | $SAHGLE= \frac{\sum_{i=1}^{N_{g}} \sum_{j=1}^{N_{s}} \frac{\mathbf{P}(i,j)i^{2}}{j^{2}}}{N_{z}}$ | Proportion of the joint distribution of smaller size zones with higher gray-level values |
| Large area low gray level emphasis | $LALGLE= \frac{\sum_{i=1}^{N_{g}} \sum_{j=1}^{N_{s}} \frac{\mathbf{P}(i,j)j^{2}}{i^{2}}}{N_{z}}$ | Proportion of the joint distribution of larger size zones with lower gray-level values |
| Large area high gray level emphasis | $LAHGLE= \frac{\sum_{i=1}^{N_{g}} \sum_{j=1}^{N_{s}} \mathbf{P}(i,j)i^{2}j^{2}}{N_{z}}$ | Proportion of the joint distribution of larger size zones with higher gray-level values |

$N_{g}$ Number of discrete intensity values in the image

$N_{s}$ Number of discrete zone sizes in the image

$N_{z}$ Number of zones in the ROI

$\mathbf{P}(i,j)$ Size zone matrix

$p(i,j)$ Normalized size zone matrix

**References**

1. Thibault G, Fertil B, Navarro C, Pereira S, Cau P, Levy N et al. Shape and texture indexes application to cell nuclei classification. *Int J Pattern Recognit Artif Intell* 2013; **27**: 1357002.

2. Van Griethuysen JJM, Fedorov A, Parmar C, Hosny A, Aucoin N, Narayan V et al. Computational radiomics system to decode the radiographic phenotype. *Cancer Res* 2017; **77**: e104–e107.
